# The Control of Super-Normal Arterial Pressure

**Published:** 1907-06-08

**Authors:** 


### The Hospital

A Journal of

The Medical Sciences and Hospital Administration.

New Series. No. 10, Vol. I. [No. 1082, Vol. XLII.] SATURDAY, JUNE 8, 1907.

### THE CONTROL OF SUPER-NORMAL ARTERIAL PRESSURE.

The invention of various forms of apparatus by which the arterial pressure can be measured under the ordinary conditions of clinical practice, has caused a wide appreciation of the influence exercised by blood-pressure in the production of pathological disturbances and diseased processes. Possibly there is at present a tendency to over-emphasise the prevailing fashion, and it may be that the various instruments do not record quite so accurate a statement of the circulatory condition as is claimed for them. Still there can be no question that the movement by which the clinician is able to approximate his observations to the precise method of the scientific laboratory is a distinct gain to practical medicine. Among the various workers in this department the name of Dr. George Oliver well deserves honourable mention, and the fact that his patient and ingenious observations are the products of clinical rather than laboratory investigations will not lessen his authority with the practising physician. After all, at the bedside of the individual patient there are conditions which cannot be reproduced in the laboratory. Dr. Oliver's work has been pursued, mainly at least, in the exact circumstances which occur in practice, and for that reason, as well as for its essential merit, it demands careful attention. In his latest paper on the subject, read a few weeks ago before the Therapeutical Society, he deals with the methods by which excessive arterial pressure may be kept in check, and shows how important is the scope of treatment in this direction.

First, on the question of diet in cases of abnormally high blood-pressure. Here evidence is distinct that a reduction of quantity as well as a modification of quality is needed. The meals should be small, and those foods which stimulate cardio-vascular activity should be taken in strict moderation. There is a general agreement that flesh meat falls among this number, and for the most part the indictment is urged against the "red" rather than the "white" meats. But Dr. Oliver finds that all of them—beef, mutton, fish, poultry, etc.—come under much the same condemnation so long as they are roasted and taken with gravy. The method of boiling rather than of roasting is to be preferred, and meat extractives, such as soups and beef teas, ought to be entirely excluded from the diet. The use of green vegetables and of fruits, on the other hand, is to be encouraged, and carbohydrate foods may be taken in moderation. The diet, in short, should be of an ordinary plan on a reduced scale, with a somewhat smaller proportion of animal and a larger proportion of vegetable foodstuffs than is common. It is satisfactory to have this statement advanced on the basis of exact measurements made under clinical conditions. So many dietetic schemes have been urged on what have been claimed to be scientific reasons, and have proved themselves in practice to be unsatisfactory, that not a few practitioners refuse to listen to any discussion on the specific values of foodstuffs outside the teachings of practical experience. Here, however, are results which stand on a very different footing, and though they agree generally with common opinion, they show the necessity of modifying this in certain detailed directions.

Next to foods comes the question of beverages and condiments, and here again Dr. Oliver's measurements oppose the adoption of extreme courses. Fluids, and especially soft non-aerated water, may be taken freely, by preference, when the stomach is empty—that is night and morning or before meals. Tea and coffee are among the favourite victims of the faddist, but provided that they are not taken in excess, and do not cause obvious ill-effects to the individual patient, there is no reason to exclude them. Alcohol, as might be expected, is found to be generally inadvisable in cases of high blood-pressure, though in cases in which the measurement is only slightly above the normal, small quantities are not found to be injurious. As regards the use of chloride of sodium, so much has recently been said as to the injurious effects of this in renal inadequacy that it is satisfactory to know that Dr. Oliver has carefully studied its relation to cases in which, without appreciable organic changes in the kidney, there is an abnormal level of blood-pressure. He does not find any distinct claim for the prescription of a strict chloride-free diet in these cases, though as a temporary measure, when accessions of pressure arise, it may be adopted with advantage. In this connection it is necessary to note that to obtain such a diet the mere omission of salt as a condiment is not sufficient; the selected foods themselves must be those naturally free from sodium chloride. Practically, a satisfactory diet scheme for such a purpose may be found in vegetable soups (made without meat stock), fresh green vegetables, fruits, nuts, fats (fresh butter, cream), salt-free bread, farinaceous vegetables (potatoes, rice, peas), and sugar. This, as already said, may meet a temporary aggravation of high pressure, but, other reasons apart, it is too insipid a programme to be continued for more than a week or two. Of the value of rest, exercise, electricity, and baths, Dr. Oliver has much to say that is both valuable and interesting, but space forbids its discussion here. Speaking generally, his conclusions are in harmony with the practice of most men of moderate opinions, though they correct these on certain points of detail. And they have the additional value that they are based, not upon empiricism, but on serious physiological and clinical study.

Regarding drugs, Dr. Oliver does not hesitate to say that they are advisable or necessary in most cases. Evacuants and intestinal antiseptics have an assured position, but some of the other observations are of decided interest. Among these may be mentioned the capacity of thyroid gland and of salts of hippuric acid to reduce abnormal blood-pressure, and the conclusion that potassium iodide has no efficacy in this direction. This last remark contradicts many clinical statements, but it is in entire harmony with the verdict of pharmacological experiment.

